# Preoperative risk factors for massive blood loss in adrenalectomy for pheochromocytoma

**DOI:** 10.18632/oncotarget.20396

**Published:** 2017-08-22

**Authors:** Hongju Liu, Bin Li, Xuerong Yu, Yuguang Huang

**Affiliations:** ^1^ Department of Anesthesiology, Peking Union Medical College Hospital, Beijing, China; ^2^ Department of Anesthesiology, Jiujiang Maternal and Child Care Centres, Jiangxi, China

**Keywords:** adrenalectomy, pheochromocytoma, risk factors, massive blood loss

## Abstract

**Background:**

This retrospective analysis of patients who underwent adrenalectomy for pheochromocytoma aimed to determine preoperative risk factors for intraoperative massive blood loss. Preoperative identification of patients at high-risk of massive blood loss may be helpful in anesthesia management and preoperative preparation.

**Materials and Methods:**

The study involved data of 268 patients who had undergone pheochromocytoma surgery at the Peking Union Medical College Hospital between January 1, 2013 and October 31, 2016. For analysis, the patients were grouped according to intraoperative blood loss: ≥ 20% of estimated blood volume (group A, *n =* 38) and < 20% of estimated blood volume (group B, *n =* 230). Perioperative characteristics were compared between the two groups. Significant variables were selected for a forward stepwise binary logistic regression analysis to determine the independent risk factors for massive blood loss.

**Results:**

The two groups showed significant differences in tumor location, tumor size, operative approach, preoperative 24-hour urine level of total noradrenaline, preoperative hemoglobin concentration, phenoxybenzamine maximum daily dose, preoperative preparation time, intraoperative urine volume, crystalloid and colloidal fluid volumes, allogeneic red blood cell transfusion, plasma and autologous blood transfusion volumes, incidence of prolonged hypotension, postoperative drainage volume, lowest and discharge hemoglobin concentrations, length of stay in intensive care unit and length of postoperative hospitalization. Binary logistic regression analysis indicated increased risk of intraoperative massive blood loss in subjects with tumors proximal to vessels or other organs (odds ratio (OR): 4.227), with tumors ≥ 5 cm (OR: 7.321), or with preoperative preparation time of ≤ 14 days (OR: 17.747).

**Conclusions:**

Tumors proximal to vessels and other organs or with maximum diameter of ≥ 5 cm (as shown by preoperative radiographic evidence), and preoperative preparation time of ≤ 14 days were independent risk factors of intraoperative massive blood loss in patients treated with adrenalectomy for pheochromocytoma.

## INTRODUCTION

Pheochromocytoma is a rare tumor, with 80–85% of cases derived from the chromaffin cells of the adrenal medulla. Its pathogenesis is complicated and not fully understood [1, 2], but it is known that the high amounts of catecholamines secreted by pheochromocytomas can lead to continuous or paroxysmal arterial hypertension [3]. Currently, the only curative therapy for pheochromocytoma is surgical removal by adrenalectomy. However, compared with adrenalectomy for other types of adrenal tumors, adrenalectomy for pheochromocytoma requires longer operative time and is associated with a higher amount of blood loss [4, 5].

Massive blood loss usually occurs during the operative steps of tumor dissection and vessel ligation. The sudden decrease in plasma catecholamine concentration, upon tumor removal, and simultaneous bleeding lead to inadequate circulating blood volume, with the potential to manifest life-threatening prolonged hypotension. Cases with tumors of large size or proximal to vessels and other organs can experience operative blood loss reaching thousands of milliliters [6] and have reported blood transfusion rates up to 10% [7].

Due to concerns about the potential risks described above, additional costs, and availability of allogeneic blood for transfusion, several blood conservation modalities have been developed, including preoperative autologous blood donation and erythropoietin administration, acute normovolemic hemodilution, and intraoperative use of blood salvage and antifibrinolytic drugs. The implementation of such modalities perioperatively has already reduced the transfusion requirement during surgery. Yet, the cases remain costly and not risk-free. It is important to apply these modalities to patients who are at high-risk for massive blood loss and blood transfusion, and to determine the most effective way of identifying such patients as they represent the most likely to benefit from the added cost, operative time and related risks associated with the blood conservation modalities themselves.

Identification of risk factors predictive of intraoperative massive blood loss before elective surgery will allow for targeted use of blood conservative modalities, and assist in effective preoperative vasoactive drugs preparation, thereby improving patient safety during adrenalectomy for pheochromocytoma. Previous studies have indicated that tumor size [8], location [6] and functional status [4] may increase the risk of intraoperative blood loss during adrenalectomy. A few studies have also characterized the preoperative risk factors for intraoperative massive blood loss in pheochromocytoma surgery.

The aim of this study was to retrospectively investigate risk factors for massive blood loss in adrenalectomy for pheochromocytoma, with the intent for the findings to assist surgical teams in preoperative vasoactive drugs preparation and blood conservation technique use.

## RESULTS

A total of 268 patients were identified in our hospital’s medical records as having undergone pheochromocytoma surgery and experienced intraoperative blood loss at the Peking Union Medical College Hospital between January 1, 2013 and October 31, 2016. These patients included 38 (14.18%) with ≥ 20% loss of the estimated blood volume (group A) and 230 (85.82%) with < 20% loss of the estimated blood volume. The 20% estimated blood volume was similar for both groups (group A: 881.45 ± 152.33 mL vs. group B: 924.49 ± 168.14 mL, *p* = 0.514). But, group A showed significantly higher estimated total intraoperative blood loss than group B (1450.00 (1000.00, 2500.00) mL vs. 100.00 (50.00, 300.00) mL, *p* = 0.000).

A total of 259 (96.6%) patients underwent laparoscopic adrenalectomy, including 238 (91.9%) performed by the retroperitoneal approach and 21 (8.1%) by the trans-peritoneal approach. No case necessitated conversion to open surgery. Twenty-seven (10.1%) of the patients were diagnosed with bilateral pheochromocytomas, and all received one-stage bilateral adrenalectomy.

The overall patient demographic data, preoperative medical history, preoperative tumor radiographic reports, laboratory information, preoperative medication and planned operative approach are presented in Table 1, according to group assignment. There were significant intergroup differences for tumor location, tumor size, operative approach, preoperative 24-hour urine level of total noradrenaline, preoperative hemoglobin concentration, maximum phenoxybenzamine daily dose, and preoperative preparation time (defined as the total days of oral medication administration prior to surgery).

**Table 1:** Preoperative variables of patients undergoing adrenalectomy for pheochromocytoma (*n* = 268)

Variable	Group A (*n* = 38)	Group B (*n* = 230)	*p*
Sex Male Female	15 (39.5) 23 (60.5)	112 (48.7) 118 (51.3)	0.292
Age, in years	43.29 ± 13.86	45.77 ± 15.60	0.358
Height, in cm	163.55 ± 8.60	165.55 ± 7.95	0.157
Weight, in kg	62.95 ± 10.88	66.03 ± 12.01	0.140
Body mass index	23.45 ± 2.91	23.98 ± 3.24	0.350
Coexisting diseases No Yes	35 (92.1) 3 (7.9)	211 (91.7) 19 (8.3)	0.939
Tumor number Single Multiple	36 (94.7) 2 (5.3)	205 (89.1) 25 (10.9)	0.288
Tumor location Unremarkable Proximal to vessels/organs	18 (47.4) 20 (52.6)	184 (80.0) 46 (20.0)	0.000*
Radiographic tumor size, as maximum diameter in cm	7.32 ± 3.01	4.42 ± 1.75	0.000*
Operative approach Laparoscopic Open	32 (84.2) 6(15.8)	227 (98.7) 3 (1.3)	0.000*
Prior retroperitonium operation No Yes	37 (97.4) 1 (2.6)	223 (97.0) 7 (3.0)	0.890
24-h urine NE, in µg	163.33 (51.61, 311.22)	83.57 (25.25, 221.40)	0.043*
24-h urine E, in µg	2.78 (1.26, 4.55)	3.17 (2.11, 5.30)	0.438
24-h urine DA, in µg	184.44 (100.00, 236.23)	166.71 (122.09, 256.32)	0.381
Hemoglobin, in g/L	128.92 ± 17.28	135.92 ± 16.08	0.015*
Platelet count, as ×10^9^/L	254.61 ± 84.35	247.87 ± 84.25	0.648
Prothrombin time, in s	11.64 ± 0.86	11.53 ± 0.70	0.394
Activated partial thromboplastin time, in s	28.93 ± 11.17	26.85 ± 4.00	0.262
International normalized ratio	1.01 ± 0.07	0.99 ± 0.07	0.209
Fibrinogen, in mg/dL	2.90 ± 0.99	2.97 ± 1.12	0.675
Phenoxybenzamine dose, in mg/d	30.00 (20.00, 30.00)	20.00 (15.00, 30.00)	0.000*
Preparation time, in d	20.00 (7.00, 30.00)	30.00 (25.75, 36.00)	0.014*
Medication other than phenoxybenzamine No Yes	31(81.6) 7(18.4)	208(90.4) 22(9.6)	0.104

Data are presented as *n* (%), mean ± SD, or mean (25th and 75th percentiles). *p < 0.05.

DA, dopamine; E, epinephrine; NE, norepinephrine.

The intraoperative management records and postoperative results of the total patients are listed in Table 2, according to group assignment. Intraoperative prolonged hypotension was defined as 30 or more consecutive minutes of hypotension (mean arterial pressure of < 60 mmHg) or requirement for vasopressor medications to maintain the blood pressure [9]. Patients in group A showed greater urine output, higher incidence of prolonged hypotension, and greater volumes of crystalloid and colloid fluids, allogeneic and autologous red blood cells, and fresh-frozen plasma intraoperatively, as well as more postoperative drainage. When compared to baseline values, the levels of postoperative and discharge hemoglobin were lower for both group A and B patients (*p* = 0.000). Patients in group A showed longer postoperative stay in the intensive care unit as well as the hospital than patients in group B.

**Table 2:** Intraoperative and postoperative results (*n* = 268)

Variable	Group A (*n* = 38)	Group B (*n* = 230)	*p*
Urine output, in mL	900.00 (600.00, 1425.00)	400.00 (200.00, 700.00)	0.000*
Crystalloids, in mL	3550.00 (2700.00, 4700.00)	2500.00 (2000.00, 3000.00)	0.000*
Colloids, in mL	1500.00 (1375.00, 2625.00)	1000.00 (500.00, 1000.00)	0.000*
Allogeneic RBC, in U	4.00 (0.00, 6.00)	0.00 (0.00, 0.00)	0.000*
Autologous RBC, in mL	0.00 (0.00, 566.50)	0.00 (0.00, 0.00)	0.000*
Fresh frozen plasma, in mL	0.00 (0.00, 400.00)	0.00 (0.00, 0.00)	0.000*
Postoperative drainage, in mL	300.00 (231.25, 695.00)	153.50 (80.00, 258.00)	0.000*
Postoperative lowest hemoglobin, in g/L	93.50 (86.00, 101.00)	109.50 (99.75, 120.00)	0.000*
Hemoglobin at discharge, in g/L	102.55 ± 12.72	116.71 ± 16.42	0.000*
Intraoperative prolonged hypotension Yes No	3/7.9 35/92.1	0/0 230/100	0.000*
Postoperative stay in ICU, in d	2.00 (2.00, 200)	2.00 (2.00, 2.00)	0.000*
Postoperative stay in hospital, in d	7.50 (6.75, 10.25)	6.00 (5.00, 7.00)	0.000*

Data are presented as *n*/%, mean ± SD, or mean (25th and 75th percentiles). *p < 0.05

ICU, intensive care unit; RBC, red blood cells.

Binary logistic regression analysis was applied to those preoperative variables that showed statistically significant differences between the two groups. Tumor proximity to vessels or other organs, tumor size and preoperative preparation time were found to be independent risk factors for massive blood loss in adrenalectomy for pheochromocytoma.

Previous studies have used 4 cm [10], 5 cm [11], 6 cm [12, 13] or 8 cm [14] as cutoff points for similar analyses. For clinical convenience, our hospital had routinely defined tumors with maximum diameter of ≥ 5 cm giant adrenal tumors; therefore, 5 cm was set in this study as the cutoff point for tumor size. Furthermore, the median preoperative time for our study population was 14 days, which was then set as the cutoff point of preoperative preparation time. These two continuous variables were transformed to categorical variables according to their cutoff points, and designated as corrected tumor size ≥ 5 cm and preoperative preparation time of ≤ 14 days for comparison between the two groups (Table 3).

**Table 3:** Transformed preoperative variables of patients undergoing adrenalectomy for pheochromocytoma (*n* = 268)

Variable	Group A (*n* = 38)	Group B (*n* = 230)	*p*
Tumor size, in cm ≥ 5 < 5	32 (84.2) 6 (15.8)	77 (33.5) 153 (66.5)	0.000*
Preoperative preparation time, in d > 14 ≤ 14	21 (55.3) 17 (44.7)	220 (95.7) 10 (4.3)	0.000*

Data are presented as *n*(%). *p < 0.05.

Tumor location, along with the two transformed variables, were included in binary logistic regression analysis. As shown in Table 4, relative to the controls, there was increased risk of massive intraoperative blood loss in patients with tumors that were either proximally located to vessels or other organs (odds ratio (OR): 4.227) or *≥* 5 cm in size (OR: 7.321), or in patients who had preoperative preparation time of ≤ 14 days (OR: 17.747).

**Table 4:** Results of binary logistic regression analysis for preoperative variables with massive blood loss (*n* = 268)

Variable	β	SE	*p*	OR	95%CI
Tumor located proximal to vessels or other organs	1.441	0.459	0.002	4.227	1.718–10.398
Tumor size of ≥ 5 cm	1.991	0.504	0.000	7.321	2.726–19.665
Preoperative preparation time of ≤ 14 d	2.876	0.560	0.000	17.747	5.918–53.224

## DISCUSSION

The greater intraoperative blood loss that occurs in pheochromocytoma surgery, as compared to surgeries of other adrenal tumors, is due to the abundant blood supply and excessive release of catecholamines of these particular tumors [4, 5]. In our study, the patients’ average blood loss (491 (50–13500) mL) was remarkably greater than that reported from previous research studies (117–181 mL) [4, 7, 15]. If our study had excluded the patients with massive bleeding (group A, representing 14% of the total cases), the average blood loss would have been only 198 mL, similar to the amounts reported in the previous articles. When the massive bleeding group (group A) was compared to the other patients in our study (group B, serving as control), the former had greater transfusion requirement for allogeneic red blood cells and fresh frozen plasma, and more frequently experienced prolonged intraoperative hypotension and required vasoactive drug infusion to maintain circulatory perfusion (3 cases in group A).

Major *et al.* [16] suggested that it is not necessary to perform routine drainage after scheduled uncomplicated laparoscopic adrenalectomy. However, considering the characteristics of pheochromocytoma, all of our patients had a drain inserted in the operational field. When we assessed the drainage features between our two groups, we determined that patients in group A had significantly more postoperative drainage. Moreover, the group A patients had longer postoperative stays in both the intensive care unit and hospital.

Accurate prediction of intraoperative blood loss will help clinical staff to optimize blood conservation protocols (e.g., preoperative autologous blood donation, acute normovolemic hemodilution, intraoperative use of blood salvage and antifibrinolytic drugs) and fully prepare allogeneic blood products as well as vasoactive drugs to ensure patient safety. In our study, we found that tumors proximally located to vessels or other organs or with size of ≥ 5 cm, and preoperative preparation time of ≤ 14 days were predictors of massive blood loss in adrenalectomy for pheochromocytoma.

When tumors were proximal to surrounding vessels or other organs, there was high risk for injury to the vessels or organs during the tumor resection process, and therefore increased risk of intraoperative bleeding. The normal location of the adrenal glands, itself, lends potential to a pheochromocytoma being attached to the inferior vena cava, renal artery, and vein, liver or pancreas [6]. Injury to such vascular structures and organs may also occur during the tumor resection, with lacerations capable of inducing intraoperative massive bleeding. In our study, those patients who had radiographic reports of “tumor in proximity to vessels or other organs” showed risk of intraoperative massive bleeding that was 4.227 times higher than for the other patients. This finding supports the recommendation of advance preparation, as described above, for such patients.

Theoretically, resection of tumor is always accompanied by dissection along the surface, which is accompanied by bleeding of the exposed surface and tissues that requires effective targeted coagulation techniques. Therefore, the larger a tumor is, the more surface is to be exposed, and the more blood will be lost. Natkaniec *et al.* [17] reported 530 cases of laparoscopic adrenalectomy and found that intraoperative blood loss was significantly greater in cases of tumor with diameter ≥ 6 cm. Yet, studies on the impact of adrenal tumor size on intraoperative blood loss have yielded inconsistent results. As reported by Agrusa *et al.* [12], and other authors [13, 18, 19], tumor size does not correlate with blood loss. In our study, however, we found that in cases of tumor with maximum diameter of ≥ 5 cm, the risk of intraoperative massive blood loss was increased by 7.321 times. This finding in our study might be explained by the design of the previous studies, which included assessment of adrenal tumors in general (compared to our focus on pheochromocytoma).

We noted that pheochromocytoma with a more prominent network of vessels tended to bleed more during resection; therefore, tumor size had more robust impact on intraoperative bleeding. Resection of other adrenal tumors that did not feature prominent vessel networks was successfully completed. The treating surgeons for these cases had skilled expertise and the patients had adequate hemostasis during the procedure, indicating that tumor size was not a meaningful contributing factor to the intraoperative bleeding. It is also important to note that greater diameter tumors may cause higher elevation of catecholamine level and hypertension frequency and extent [4, 18]; the higher pressure could in turn cause greater bleeding during tumor resection.

In our study, we found that the risk of massive blood loss was increased by 17.747 times when the patients’ preoperative preparation time was ≤ 14 days. It is generally accepted in today’s medical practice that initial therapy is introduced 10–14 days prior to surgery [20], helping to ensure that patients receive appropriate preoperative optimization with stable hypertension control and adequate blood volume expansion. Inadequately prepared patients may develop hypertensive crisis and widely fluctuating blood pressure, which may cause increased bleeding during the surgery. The findings from the current study indicate that the clinical care team should take precautions to ensure satisfactory preparation of pheochromocytoma patients, in order to reduce the risk of intraoperative bleeding.

Our study has some limitations that must be taken into consideration when interpreting our results. We found only three independent risk factors of intraoperative massive blood loss in adrenalectomy for pheochromocytoma, which might help in decision-making for using blood conservation techniques. A predictive model, therefore, would be more helpful in clinical practice to identify patients undergoing adrenalectomy for pheochromocytoma who are at risk of massive bleeding. Further studies with more samples are needed. Meanwhile, the nature of a single-center study limits its external validity, and multicenter trials will be necessary to confirm our findings.

In conclusion, patients with tumors proximal to vessels and other organs or with maximum diameter of ≥ 5 cm, and preoperative preparation time of ≤ 14 days have increased risk of massive blood loss in adrenalectomy for pheochromocytoma. This knowledge may contribute to improved preoperative preparation, involving volume management, vasoactive drugs and blood conservation strategy.

## MATERIALS AND METHODS

### Patient population

After institutional ethics approval was obtained, data of patients who underwent elective pheochromocytoma surgery between January 1, 2013, through October 31, 2016 at the Peking Union Medical College Hospital were obtained. During the targeted study period, 272 patients underwent adrenalectomy for pheochromocytoma; of those, 268 cases were selected for study inclusion, with 4 cases being excluded due to incomplete data. Data for each study patient were collected from our Hospital Information System (known as HIS) and operation-anesthesia databases, and included sex, age, height, weight, preoperative medical history, radiographic reports of tumor, laboratory information, preoperative medication, operative approach, intraoperative fluid management, hemoglobin concentration, intra- and postoperative blood loss, pathology report, complications, and length of stay in the intensive care unit and hospital.

In our hospital, all the pheochromocytoma patients received preoperative preparation according to the following protocol: 1) oral phenoxybenzamine administration until the day before surgery, with dose adjusted to ensure blood pressure < 120/80 mmHg when sitting and systolic blood pressure > 90 mmHg when standing; 2) oral metoprolol administration until the day before surgery in the case of concurrent tachycardia, and dose adjustment to ensure heart rate of 60∼70 bpm when sitting and of 70∼80 bpm when standing; 3) encouragement for oral fluid intake, to promote decreased hematocrit and increased body weight, among other benefits; and 4) perform any necessary care to optimize cardiac function and treat the symptoms of high metabolic syndrome and impaired glucose tolerance. Surgery was arranged after hemodynamic stability was achieved. All surgeries were conducted by urological attending surgeons, each with at least 3 years experience in performing adrenalectomy for pheochromocytomas. The intraoperative blood loss was calculated as the sum of the blood volume collected in the suction container and the soaked sponge.

Guidelines suggest autologous blood transfusion when estimated blood loss is ≥ 20% of estimated blood volume [21]. Estimated blood volume (in mL) was calculated as body weight (in kg) * 0.07 * 1000. The patients selected for study inclusion were retrospectively divided into the massive bleeding group (group A, with intraoperative total blood loss of ≥ 20% of the estimated blood volume, *n* = 38) and the control group (group B, with intraoperative total blood loss < 20% of the estimated blood volume, *n* = 230).

### Statistical analysis

Data were analyzed with standardized statistical software (Statistical Package for the Social Sciences, version 17.0; SPSS, Inc., Chicago, IL, USA). Categorical variables were summarized as frequency. Continuous variables were summarized as mean and standard deviation if normally distributed, or as median and 25th and 75th percentiles if not normally distributed. Characteristics of the two groups were compared by using the *t*-test or the Wilcoxon’s rank-sum test for continuous variables and the chi-square test for categorical variables. Intragroup comparison was made by using the paired *t*-test. A *p*-value of < 0.05 indicated statistical significance. Continuous variables with statistical significance (*p* < 0.05) were transformed to categorical variables by their cutoff points. Binary logistic regression analysis was performed to determine the independent risk factors of massive blood loss and to calculate the OR and corresponding 95% confidence intervals (CIs).
